# MiR-214 Targets β-Catenin Pathway to Suppress Invasion, Stem-Like Traits and Recurrence of Human Hepatocellular Carcinoma

**DOI:** 10.1371/journal.pone.0044206

**Published:** 2012-09-04

**Authors:** Hongping Xia, London Lucien P. J. Ooi, Kam M. Hui

**Affiliations:** 1 Bek Chai Heah Laboratory of Cancer Genomics, Division of Cellular and Molecular Research, Humphrey Oei Institute of Cancer Research, National Cancer Centre, Singapore, Singapore; 2 Department of Surgical Oncology, Singapore General Hospital, Singapore, Singapore; 3 Cancer and Stem Cell Biology Program, Duke-NUS Graduate Medical School, Singapore, Singapore; 4 Department of Biochemistry, Yong Loo Lin School of Medicine, National University of Singapore, Singapore, Singapore; 5 Institute of Molecular and Cell Biology, A*STAR, Biopolis Drive Proteos, Singapore, Singapore; Northwestern University Feinberg School of Medicine, United States of America

## Abstract

The down-regulation of miR-214 has previously been observed in human hepatocellular carcinoma (HCC). Here, we demonstrated the down-regulation of miR-214 is associated with cell invasion, stem-like traits and early recurrence of HCC. Firstly, we validated the suppression of miR-214 in human HCC by real-time quantitative RT-PCR (qRT-PCR) in 20 paired tumor and non-tumor liver tissues of HCC patients and 10 histologically normal liver tissues from colorectal cancer patients with liver metastases. Further qRT-PCR analysis of 50 HCC tissues from an independent cohort of HCC patients of whom 29 with early recurrent disease (<2 years) and 21 with late recurrent disease demonstrated that the suppression of miR-214 was significantly more suppressed in samples from HCC patients with early recurrent disease compared those from patients with no recurrence. Re-expression of miR-214 significantly suppressed the growth of HCC cells *in vitro* and reduced their tumorigenicity *in vivo*. The enhancer of zeste homologue 2 (*EZH2*) and β-catenin (*CTNNB1*) was identified as two potential direct downstream targets of miR-214 through bioinformatics analysis and experimentally validated the miRNA-target interactions with a dual-firefly luciferase reporter assay. In corroborate with this, both *EZH2* and *CTNNB1* are found to be significantly overexpressed in human HCC biopsies. Since *EZH2* can regulate *CTNNB1*, *CTNNB1* can also be an indirect target of miR-214 through *EZH2*. Silencing *EZH2* or *CTNNB1* expression suppressed the growth and invasion of HCC cells and induced E-cadherin (*CDH1*), known to inhibit cell invasion and metastasis. Furthermore, the silencing of miR-214 or overexpression of *EZH2* increased EpCAM^+^ stem-like cells through the activation of *CTNNB1*. Interestingly, the up-regulation of *EZH2*, *CTNNB1* and the down-regulation of *CDH1* in HCC patients correlated with early recurrent disease and can be an independent predictor of poor survival. Therefore, miR-214 can directly or indirectly target *CTNNB1* to modulate the β-catenin signaling pathway in HCC.

## Introduction

Hepatocellular carcinoma (HCC) is the most common type of primary liver cancer and the third leading cause of death from cancer. A variety of etiological and risk factors including hepatitis virus (HBV or HCV) infection, alcohol abuse and aflatoxin ingestion have been associated with hepatocarcinogenesis [1], [2], [3], [4]. The development of HCC is a multi-step process from chronic hepatitis, to cirrhosis, to dysplastic nodules, and to malignant tumors with various genetic and epigenetic alterations [3]. Although numerous studies have been devoted to delineate the molecular pathogenesis of HCC, the incidence and mortality of HCC has not been reduced over the past few decades. Surgery currently offers the only possibility of prolonged survival for HCC patients. Unfortunately, recurrence occurs in more than two-thirds of these patients despite initial curative intent and converts the situation to a dismal prognosis [1], [5]. It is presently a challenge to identify patients who are at high risk of early recurrence after undergoing potentially curative treatment for HCC. Various surrogate clinicopathologic features such as lymphovascular invasion, capsular invasion, satellite lesions, and tumour numbers are often used but with varying reliability reported [4]. Additionally, most HCC are diagnosed at the advanced stages when there is no effective treatment, so there is an urgent need to develop novel therapeutic strategies for the treatment of HCC [5].

MicroRNAs (miRNAs) are a class of highly conserved, small non-coding RNAs that play essential roles in the post-transcriptional regulation of gene expression through base pairing with the 3′-untranslated region (3′-UTR) of target mRNAs. Because miRNAs have been discovered to target a large proportion of mammalian genes, many studies have indicated that miRNAs play critical roles in the regulation of many biological functions and consequently, miRNAs play crucial roles in the development of many human diseases, including cancer [6], [7]. The dsyregulation of miRNAs in HCC have been reported using miRNA expression profiling studies with several miRNAs reported as enhancers (miR-30d, miR-151, miR-210) or suppressors (miR-122, let-7g, miR-29b, miR-193b, miR-194, miR-139 and miR-124) of the metastatic process [8]. While the down-regulation of miR-214 in HCC have been reported [9], [10], [11], [12], [13], its molecular roles in recurrent HCC remain largely unknown. In this study, we have characterized CTNNB1 and EZH2 as two functional downstream targets of miR-214 and to decipher the possible roles of these downstream targets in early recurrent HCC disease.

## Materials and Methods

### Tissue Specimens and Cell Cultures

Cancerous and non-cancerous liver tissues were obtained from patients who underwent partial hepatectomy as curative treatment for HCC. All tumor tissues were divided into two portions and immediately snap-frozen in liquid nitrogen. Half of the sample was stored in liquid nitrogen until further use while the other portion was stained with hematoxylin and eosin and evaluated by an independent pathologist. All cancerous tissues studied were at least 70% cancerous. All tissue samples employed in this study were approved and provided by the Tissue Repository of the National Cancer Center Singapore, in accordance with the policies of its Ethics Committee. Written informed consent was obtained from all participating patients and all clinical and histopathological data provided to the researchers were rendered anonymous [4]. The human HCC or hepatoma cell lines (HepG2, Hep3B, Huh-7, PLC/PRF/5, MHCC97-L, HCCLM3, MHCC97-H, SK-HEP-1, HLE, SNU-449 and SNU-475) were cultured in Dulbecco’s modified Eagle’s medium (DMEM) (Invitrogen, Carlsbad, CA) with 10% FBS and 100 units/mL of penicillin and 100 µg/mL of streptomycin (Invitrogen).

### RNA Extraction and Real-time Quantitative RT-PCR

Total RNA from the HCC tissue samples or cell lines was extracted using TRIzol reagent (Invitrogen). The quality and quantity of isolated total RNA was assessed using the Agilent 2100 Bioanalyzer and NanoDrop ND-1000 Spectrophotometer (Agilent, Santa Clara, CA, USA). qRT-PCR was performed as described [14], using primers listed in Table S1. For miRNA detection, the total RNA samples were polyadenylated and reversely transcribed for a two-step quantitative RT-PCR reaction using the NCode™ VILO™ miRNA cDNA Synthesis Kit and EXPRESS SYBR® GreenER™ miRNA qRT-PCR Kits (Invitrogen, CA) according to the manufacturer’s instructions. The sequence-specific forward primers for mature hsa-miR-214 and U6 internal control were CAGGCACAGACAGGCAGT (18 bps, GC = 61.12%, Tm = 61.3) and 5′- CTCGCTTCGGCAGCACA-3′, respectively. For mRNA detection, the total RNA was reversely transcribed by using SuperScript III First-Strand Synthesis System for RT-PCR (Invitrogen, CA). The qPCR were performed by using SsoFast™ EvaGreen® Supermix (Bio-Rad). The *U6* or *HPRT1* was used as the internal control. The expression level of miR-214, EZH2, CTNNB1 or CDH1 was calculated using the expression ratios miR-214/U6, EZH2/HPRT1, CTNNB1/HPRT1 and CDH1/HPRT1 (i.e. 2^–ΔCt^) [10], [15].

### Cell Viability Assay

A fragment containing human miR-214 was PCR-amplified from normal genomic DNA and subcloned into the pLL3.7 vector to get pLL3.7-Pre-miR-214 (P-miR-214) (Figure S3). Both HLE and SK-HEP-1 cells were either transfected with the pLL3.7-Pre-miR-214 (P-miR-214) or pLL3.7-control vector (P-miR-control) using the GenJet™ Plus DNA in vitro transfection reagent (SignaGen, MD). The cell viability was assessed by using MTS [3-(4,5-dimethylthiazol-2-yl)-5-(3-carboxymethoxyphenyl)-2-(4-sulfophenyl)-2H-tetra zolium] assays. Cells were seeded into 96-well plates at a density of 5×10^3^ per well (100 µL) 24h post-transfection. For the MTS assay, the CellTiter 96 aqueous one solution cell proliferation assay kit (Promega, Madison, WI, USA) was used. Briefly, at each of the desired time points (24 h, 48 h and 72 h), 20 µL of the MTS reagent was added into each well and the cells were incubated at 37°C for an additional 2 h. The absorbance was detected at 490 nm using a Wallac Victor 1420 Multilabel plate reader (PerkinElmer, San Diego, CA). Each experiment was repeated three times.

### In vitro Matrigel Invasion Assay

Cell invasiveness was assessed using BioCoat Matrigel invasion chambers (BD Biosciences, Bedford, MA) according to the guidelines. Briefly, 800 µL of the cell culture medium with 10% FBS was added to the lower chamber as a chemoattractant. The transfected HLE and SK-HEP-1 cells were resuspended in 500 µL serum-free medium and seeded onto the rehydrated insert at 24 h after transfection. After another 24 h of incubation at 37°C, any non-invading cells on the upper surface of the Matrigel membrane were gently removed using a cotton-tipped swab. The invaded cells were then fixed with 100% methanol and stained with 1% toluidine blue. The stained invasive cells on the lower surface of the membrane were photographed under an inverted light microscope with a 40× objective and quantified by manual counting in three randomly selected areas. This experiment was performed in duplicate in three independent experiments.

### Establishment of Stable HCC Cell Lines

One day before transfection, HLE and SK-HEP-1 cells were seeded onto 6-well plates at about 80% confluence. The cells were either transfected with pLL3.7-Pre-miR-214 (P-miR-214) or pLL3.7-miR-control (P-miR-control) vectors using the GenJet™ Plus DNA in vitro tranfection reagent (SignaGen, MD), according to manufacturer’s instructions. After 48 h, the cells were subcultured to 10% confluence in a medium containing 1 µg/mL of puromycin (Sigma-Aldrich, St. Louis, MO). When all cells in the non-transfected control culture were killed, antibiotic-resistant clones were picked and passaged through the medium containing puromycin. The expression of miR-214 was confirmed by real-time qRT-PCR, as described above.

### Soft Agar Colony Assay

The stably transfected cells was mixed with tissue culture medium containing 0.6% low-melting-point agarose (Sigma Sant Louise, MO), resulting in a final agar concentration of 0.3%. Then, 500 µL of the cell suspension (800 cells) was immediately plated in 24-well plates coated with 500 µL 0.6% agar in tissue culture medium and cultured at 37°C with 5% CO_2_. The plates were kept in the incubator and the number of colonies formed was counted under an inverted light microscope with a 40× objectives after 2–3 weeks. The assay was analyzed in duplicate in three independent experiments.

### Luciferase Reporter Assay

The 3′-UTR sequence of EZH2 and CTNNB1 predicted to interact with miR-214 or a mutated sequence within the predicted target sites was synthesized and inserted into the XbaI and FseI sites of the pGL3 control vector (Promega, Madison, WI) (Figure S3). These constructs were called pGL3-EZH2-3′UTR-wt or pGL3- EZH2-3′UTR –mut, pGL3-CTNNB1-3′UTR-wt or pGL3-CTNNB1-3′UTR-mut, respectively. For the reporter assay, SK-HEP-1 cells were plated onto 24-well plates and transfected with the above constructs and P-miR-214 or P-miR-control vectors using the GenJet™ Plus DNA in vitro tranfection reagent (SignaGen, MD). A Renilla luciferase vector pRL-SV50 (Promega, Madison, WI) was also co-transfected in order to normalize the differences in transfection efficiency. After 48 h, the cells were harvested and assayed using the dual-luciferase reporter assay system (Promega, Madison, WI) according to the manufacturer’s instructions. The experiment was performed in duplicate in three independent experiments.

**Figure 1:**
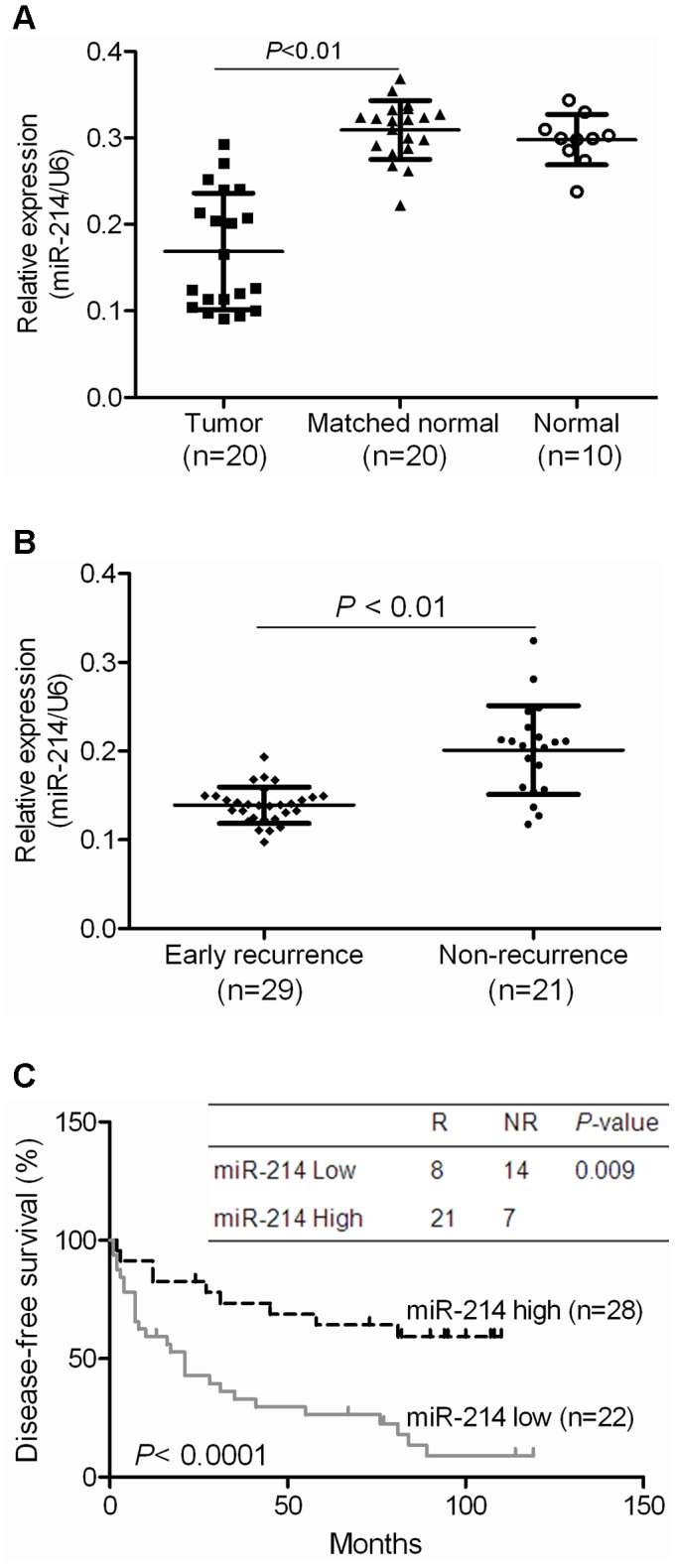
Downregulation of miR-214 is associated with the early recurrence of human HCC. The level of expression of miR-214 was analyzed by qRT-PCR and normalized to U6. (A) Expression of miR-214 in 20 paired of HCC tumor tissues was significantly lower compared to 20 matched histologically normal tissues as well as 10 histologically normal liver tissues from colorectal cancer patients with liver metastases (*P*<0.01). (B) Low miR-214 expression was associated with early recurrent HCC disease when studied in an independent cohort of 50 HCC samples. The average expression level of miR-214, analyzed by qRT-PCR, was lower in HCC patients with early recurrence (≤2 years) (n = 29) than patients with no recurrence in the same time period (n = 21). (C) The expression of miR-214 was associated with survival in patients with HCC. The median expression value obtained for miR-214 of the 50 samples studied was employed as the cut-off point. Fisher’s exact test and Kaplan-Meier analysis were used to demonstrate that high miR-214 expression was significantly associated with early recurrent disease and a relative poorer disease-free survival rate.

### Western Blotting

The protein concentrations were determined using the Bradford protein assay (Bio-Rad, Hercules, CA, USA). Antibodies for western blot: rabbit EZH2 (H-80) and rabbit beta-catenin (H-102) (Santa Cruz, CA); rabbit E-Cadherin (24E10) (Cell Signaling, MA); goat GAPDH (GenScript, NJ).

### Flow Cytometry

The stable HLE cells was transfected with anti-miR-214 construct (miRZip-214; System Biosciences, Mountain View, CA) or pLVTHM-EZH2 [16]. Transfected cells were resuspended in 100 µl staining buffer containing 10% FBS and put on ice for 20 min to block Fc receptors. After incubating with primary PerCP-Cy5.5-conjugated anti-human EpCAM antibodies or isotype control (BD Biosciences, Bedford, MA) for 1–2 h on ice in the dark, the cells were then washed with 1 ml ice-cold staining buffer for two times and centrifuged (400×g) at 4°C for 5 min. The collected cells were suspended in 500 µl staining buffer solution and were detected using FACSCanto II flow cytometer (BD Biosciences). All flow cytometry results were repeated three times.

**Figure 2:**
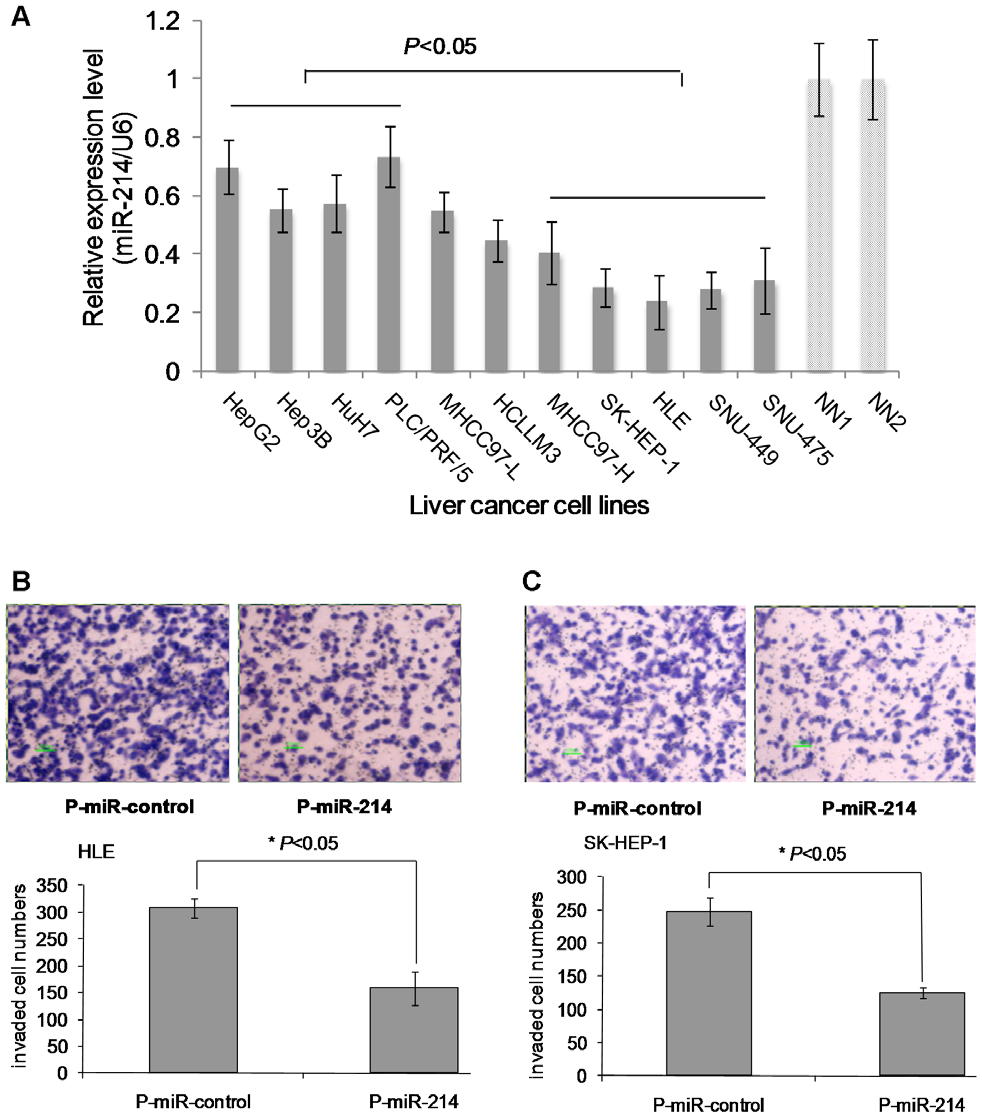
MiR-214 inhibits the invasion of HCC cells. (A) The expression of miR-214 in liver tumor cell lines was significantly lower than in the normal liver tissues (NN1 and NN2) (* *P*<0.05). (B and C) Re-expression of miR-214 following transfection with P-miR-214 inhibited the invasion of HLE (B) and SK-HEP-1 (C) cells. The upper panels in the figures showed images of transwell migration. The bar graph below the images indicated the mean number of invaded cells (± SD) counted under the microscope in three randomly selected fields (magnification ×40). *P<0.05.

### Animal Studies

All experiments on mice were approved by the SingHealth Institutional Animal Care and Use Committee (IACUC). The stably transfected SK-HEP-1 cells were resuspended in PBS and implanted into the right and left flanks (5×10^6^ cells per flank) of male BALB/c nude mice via subcutaneous injections. Tumor volumes were determined each week by measuring their length (a) and width (b) using a vernier caliper. The tumor volume (V) was calculated according to the formula V = ab^2^/2. The statistical significance between tumor sizes in the P-miR-214 and P-miR-control transfected groups was evaluated using the Student’s *t* test.

**Figure 3:**
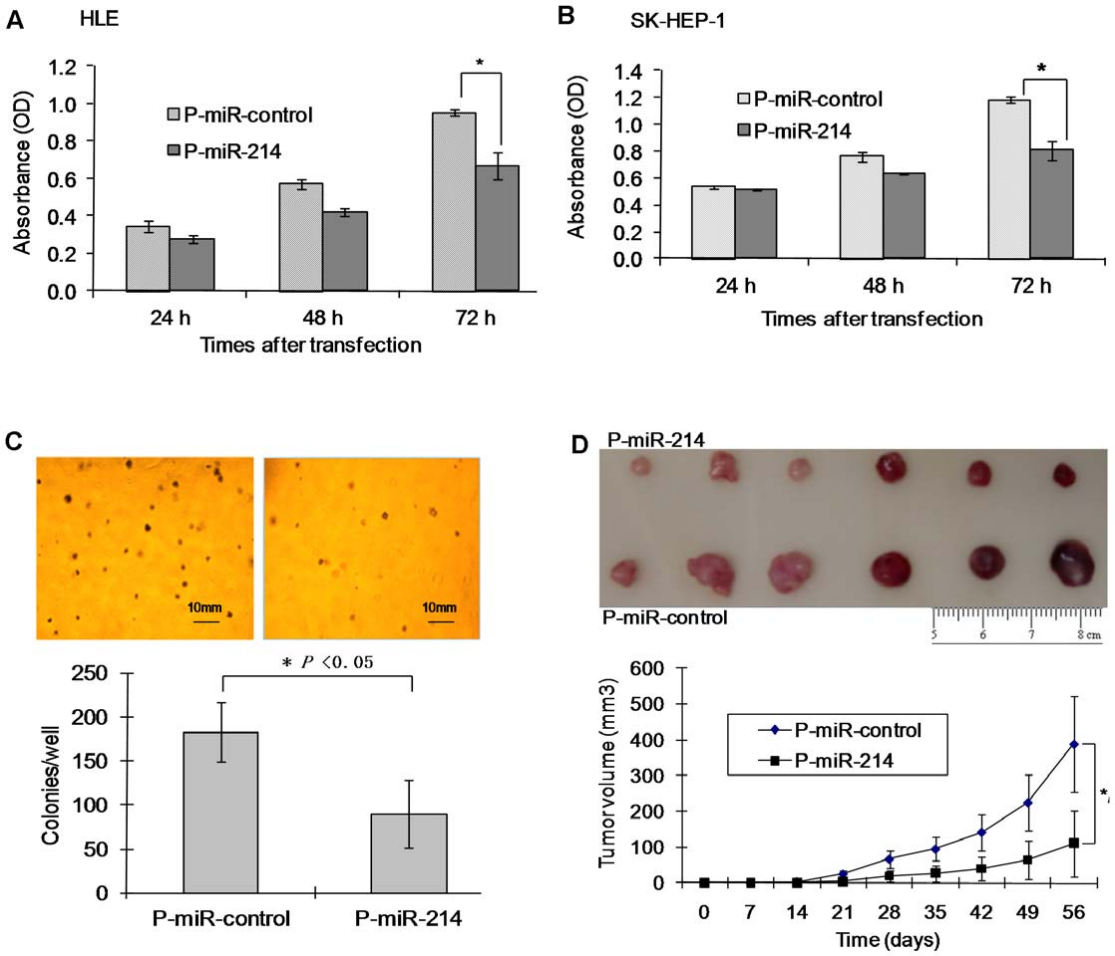
Re-expression of miR-214 significantly inhibits cell growth in vitro and tumorigenic properties of HCC cells. (A and B) Growth of HLE (A) and SK-HEP-1 (B) cells *in vitro* at different time points following the re-expression of miR-214 mediated by transfection with P-miR-214, *P<0.05. (C) Stable expression of miR-214 inhibited the anchorage-independent growth of SK-HEP-1 cells in soft agar. The upper section shows images of colony formation. The bar graph below the figures showed the mean number of colonies formed (± SD) and counted under the microscope in three randomly selected fields (magnification, ×40). *P<0.05. (D) Stable expression of miR-214 inhibited tumorigenicity of SK-HEP-1 cells. The upper section showed images of the tumors obtained in mice at the end of the eighth week. The bar graph indicated the average of overall tumor volume measured each week (n = 6 mice per group).

### Survival and Statistical Analysis

The experimental data are presented as the mean ± standard deviation (SD). All statistical analyses were performed using ANOVA or a two-tailed Student’s *t* test (GraphPad Prism 5). Disease-free survival (DFS) was measured from the date of hepatic resection to the date of recurrence within 2 years or till the last follow up. The survival curves and univariate analysis were calculated using the Kaplan-Meier method and statistically compared using a log-rank test. Any factors that were significant at P<0.05 in the univariate analysis were candidates for entry into a multivariate Cox proportional hazards model, the results of which are presented for both the first and last steps of the reverse selection of random variables. Differences were considered statistically significant when the P-values were less than 0.05.

## Results

### Downregulation of miR-214 is Associated with the Early Recurrence of Human HCC

The down-regulation of miR-214 has previously been observed in human HCC. In this study, we firstly validated the suppression of miR-214 in human HCC by qRT-PCR in paired tumor and non-tumor liver tissues from 20 HCC patients, as well as in 10 samples of histologically normal liver tissues from colorectal cancer patients with liver metastases (Fig. 1A and Figure S1). Further analysis of HCC tissues, using qRT-PCR, of an independent cohort of 50 HCC patients of whom 29 with early recurrent disease (≤2 years) and 21 with late recurrent disease demonstrated that miR-214 was significantly suppressed in samples of HCC patients with early recurrent disease, P<0.01, (Fig. 1B). Using the median expression value obtained for miR-214 of the 50 samples studied as the cut-off point, Fisher’s exact test and Kaplan-Meier analysis demonstrated that low miR-214 expression was significantly associated with early recurrent disease and a relative poorer disease-free survival rate (Fig. 1C).

**Figure 4:**
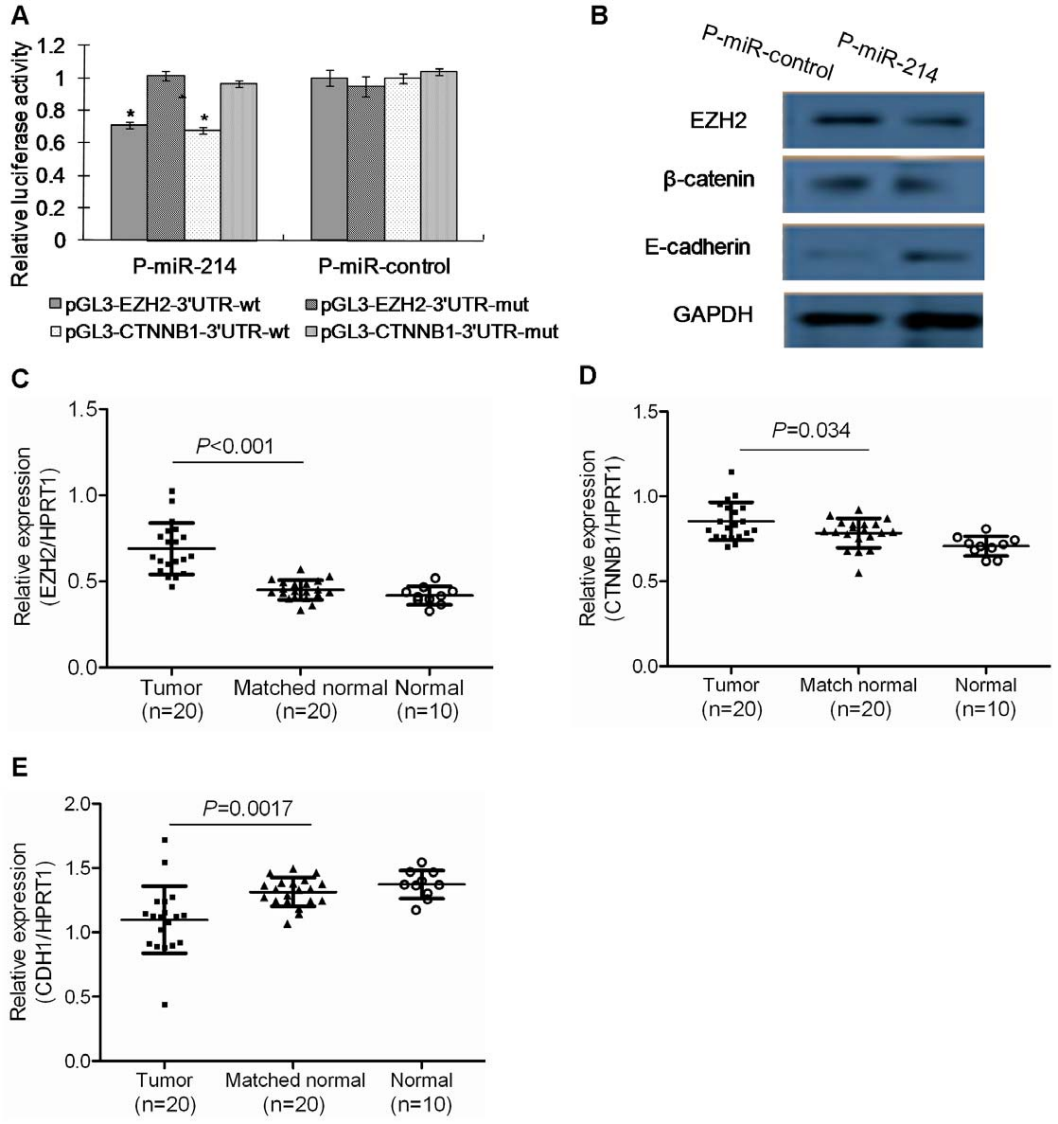
*EZH2* and *CTNNB1* are downstream targets of miR-214 and both are upregulated in human HCC tissue samples. (A) Effect of miR-214 on *EZH2* and *CTNNB1* expression, as shown by a luciferase reporter assay. The data were normalized by the ratio of Firefly and Renilla luciferase activities measured at 48 h post-transfection. The bar graph showed the mean ± SD in three independent transfection experiments. *P<0.05. (B) Western blotting analysis of EZH2, β-catenin, and E-cadherin expression in P-miR-control- and P-miR-214-transfected SK-HEP-1 cells. (C-E) Validation of the expression of *EZH2* (C), *CTNNB1* (D) and CDH1 (E) in 20 paired human HCC tissue samples and 10 samples of histologically normal liver tissues were validated by qRT-PCR.

### Re-expression of miR-214 Significantly Inhibits Cell Growth and Invasion in vitro and Tumorigenic Properties in vivo of HCC Cells

Next, we studied the expression of miR-214 in a panel of human liver cancer cell lines including HepG2, Hep3B, Huh-7, PLC/PRF/5, MHCC97-L, HCCLM3, MHCC97-H, SK-HEP-1, HLE, SNU-449 and SNU-475. Consistent with the data obtained from human HCC tissue samples, the down-regulation of miR-214 in these human liver cancer cell lines was observed (Fig. 2A). Interestingly, the expression of miR-214 was apparently more down-regulated in cell lines possess higher reported capability of invasion and metastasis (such as HLE and SK-HEP-1) than cell lines with lower invasion and metastasis capability (such as HepG2 and PLC/PRF/5) [17] (Fig. 2A). So we transfected HLE and SK-HEP-1 cells, which express miR-214 at a low level, with the pLL3.7-Pre-miR-214 (P-miR-214) or pLL3.7-miR-control vector (P-miR-control) in order to overexpress miR-214. The transfection efficiency and overexpression effects of miR-214 were shown in Figure S2. Re-expression of miR-214 in HLE and SK-HEP-1 cells expressing low basal level of miR-214 significantly reduced the invasiveness and motility ability of HLE and SK-HEP-1 cells compared to transfection with the P-miR-control (Fig. 2B and 2C).

**Figure 5:**
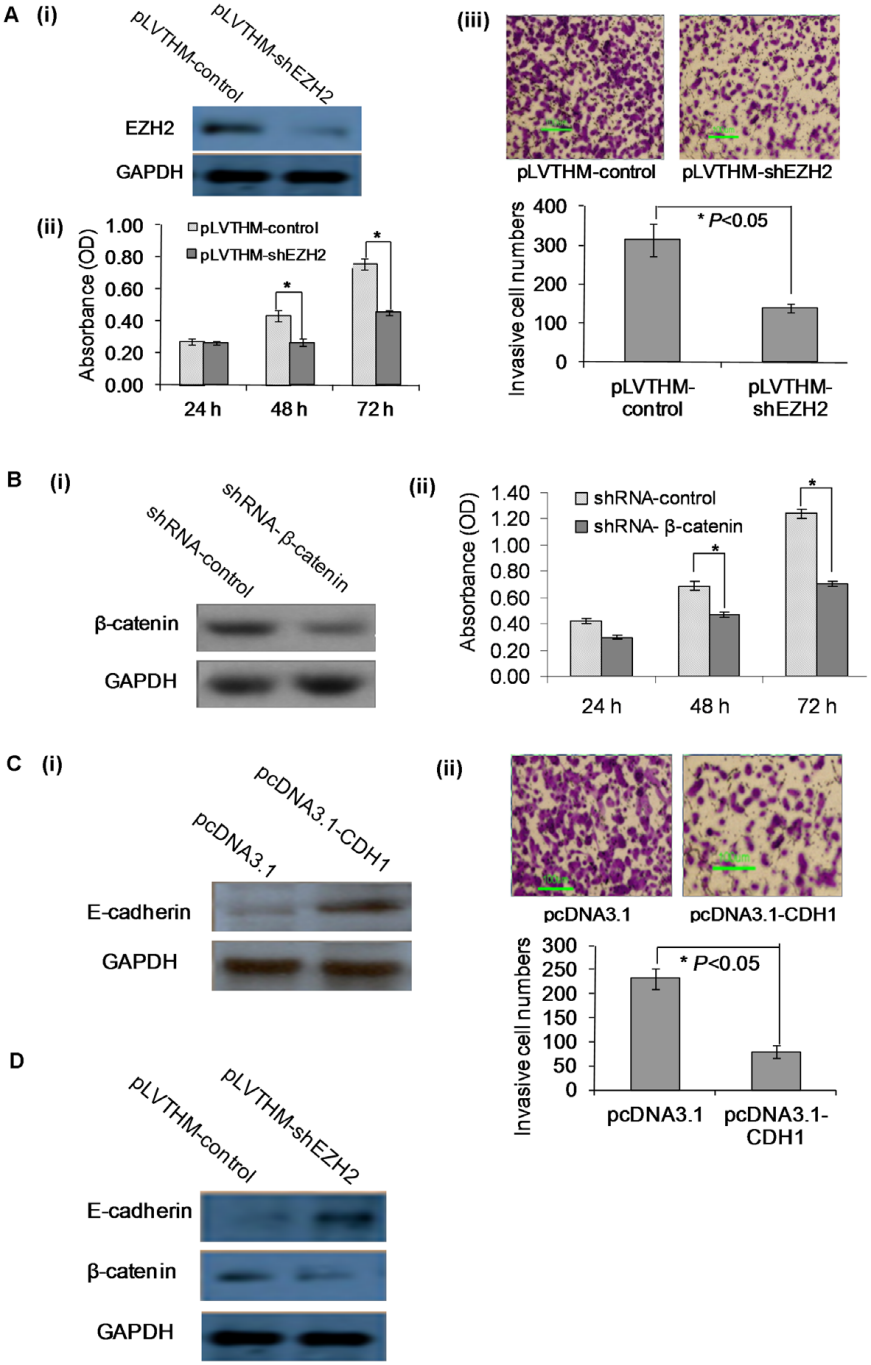
Roles of *EZH2*, *CTNNB1* and *CDH1* on the growth and invasion of HCC cells. (A) Silencing of *EZH2* significantly inhibited the growth and significant decreased the ability of SK-HEP-1 cells to invade. (i) Western blots showing the silencing of *EZH2* by pLVTHM-shEZH2. (ii) The effect of silencing *EZH2* on cell growth at different time points. (iii) The inhibitory effect of silencing *EZH2* on cell invasion. (B) Silencing of *CTNNB1* significantly inhibited the growth of SK-HEP-1 cells. (i) Western blots showing the reduction of β-catenin after transfection with shRNA-β-catenin. (ii) Effects of silencing β-catenin on cell growth at different time points. (C) Over-expression *CDH1* significantly inhibited the ability of SK-HEP-1 cells to invade. (i) Western blots showing the overexpression of CDH1 by pcDNA3.1-CDH1 plasmid transfection. (ii) The inhibitory effects of overexpressing *CDH1* on SK-HEP-1 cell invasion. (D) Western blots showing the silencing of EZH2 significantly decreased the expression of CTNNB1 and induced CDH1 expression.

Subsequently, functional overexpression of miR-214 in HLE and SK-HEP-1 cells also significantly inhibited cell proliferation according to the MTS-based CellTiter 96 cell proliferation assay (Fig. 3A and 3B) and colony formation (Fig. 3C). The MTS assay showed that overexpression of miR-214 significantly inhibited the growth of HLE and SK-HEP-1 cells at 72 h post-transfection, while P-miR-control had no effect (Fig. 3A and 3B). Moreover, stably overexpressing miR-214 in SK-HEP-1 cells resulted in the significant reduction in soft agar colony formation (Fig. 3C) indicating the overexpression of miR-214 inhibited cell anchorage-independent growth of SK-HEP-1 cells in soft agar. Furthermore, SK-HEP-1 cells stably overexpressing miR-214 following transfection with P-miR-214 (SK-miR-214) showed reduced tumorigenesis in nude mice. The mean volume of the tumors generated from SK-miR-214 cells at 8 weeks post-injection was significantly less compared to mice injected with SK-miR-control cells (Fig. 3D). These data suggested that the overexpression of miR-214 in human HCC cells can significantly inhibit their growth and invasion *in vitro* and tumorigenicity *in vivo*.

**Figure 6:**
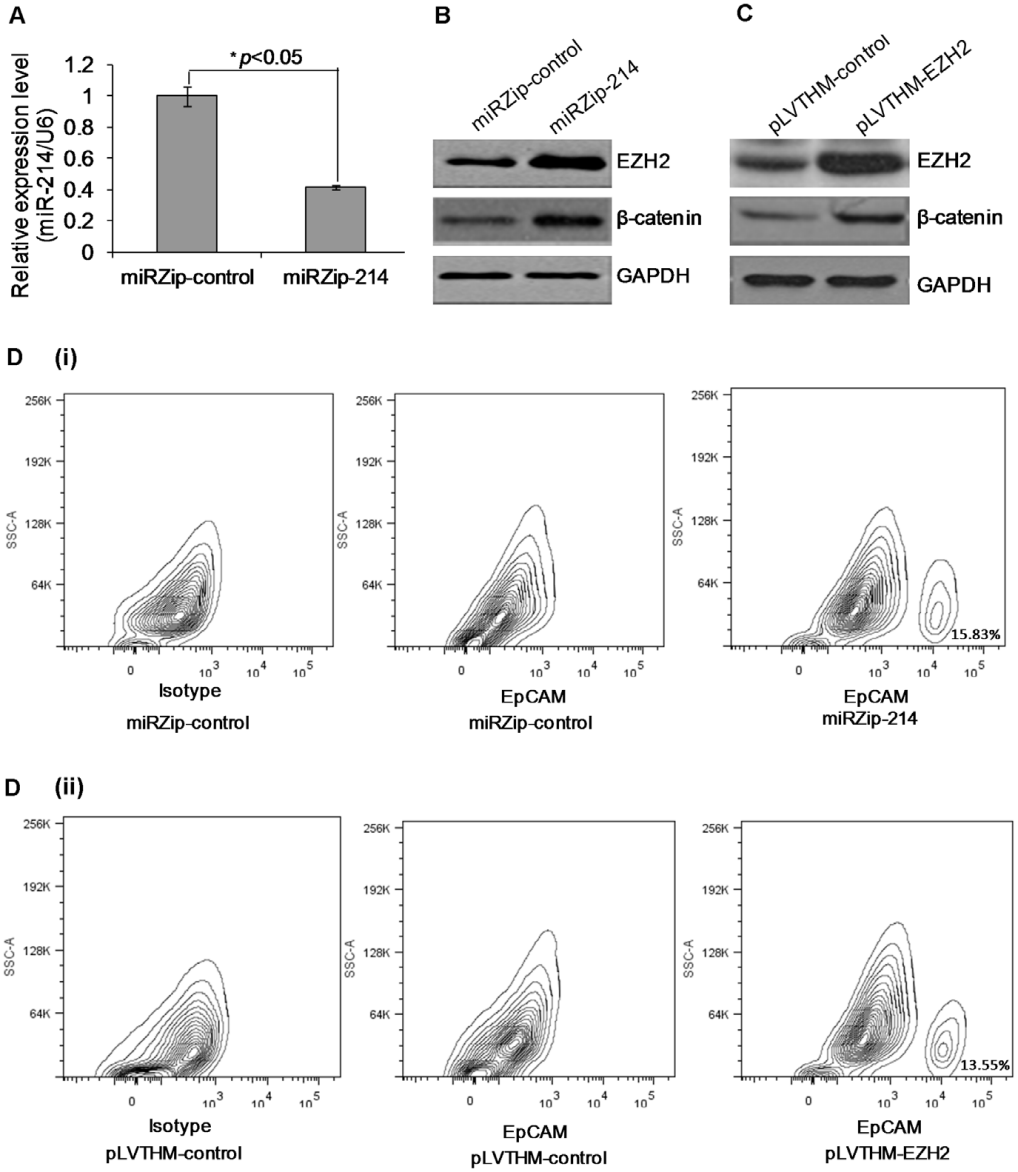
Further silencing of miR-214 in HLE cells with miRZip-214 increases EpCAM^+^ stem-like cell population by activating the β-catenin pathway. (A) Further silencing of miR-214 in HLE cells with miRZip-214. (B) Silencing miR-214 following transfection with miRZip-214 increased EZH2 and β-catenin expression. (C) Overexpression of *EZH2* with pLVTHM-EZH2 activated β-catenin expression. (D) Flow cytometric analysis of EpCAM^+^ stem-like HLE cells following the silencing of miR-214 (i) and overexpression of *EZH2* (ii).

### miR-214 can Regulate the Canonical wnt Signaling Pathway in HCC by Targeting CTNNB1, its Key Downstream Component

In order to elucidate the molecular roles of miR-214 in HCC, we have identified its potential molecular targets using computational algorithms. With an integrated target prediction tool (miRecords), *CTNNB1* was predicted to be a potential target of miR-214 by miRanda, PITA, RNAhybrid and TargetScan. Previous study has shown that the polycomb protein EZH2 was regulated by miR-214 in skeletal muscle and embryonic stem cells [18]. In addition, *EZH2* was predicted to be a potential target of miR-214 by RNAhybrid [19]. The predicted target sequences of miR-214 and EZH2-3′UTR or CTNNB1-3′UTR was analyzed by RNAhybrid 2.2 or TargetScan and these sequences are shown in Figure S3.

**Figure 7:**
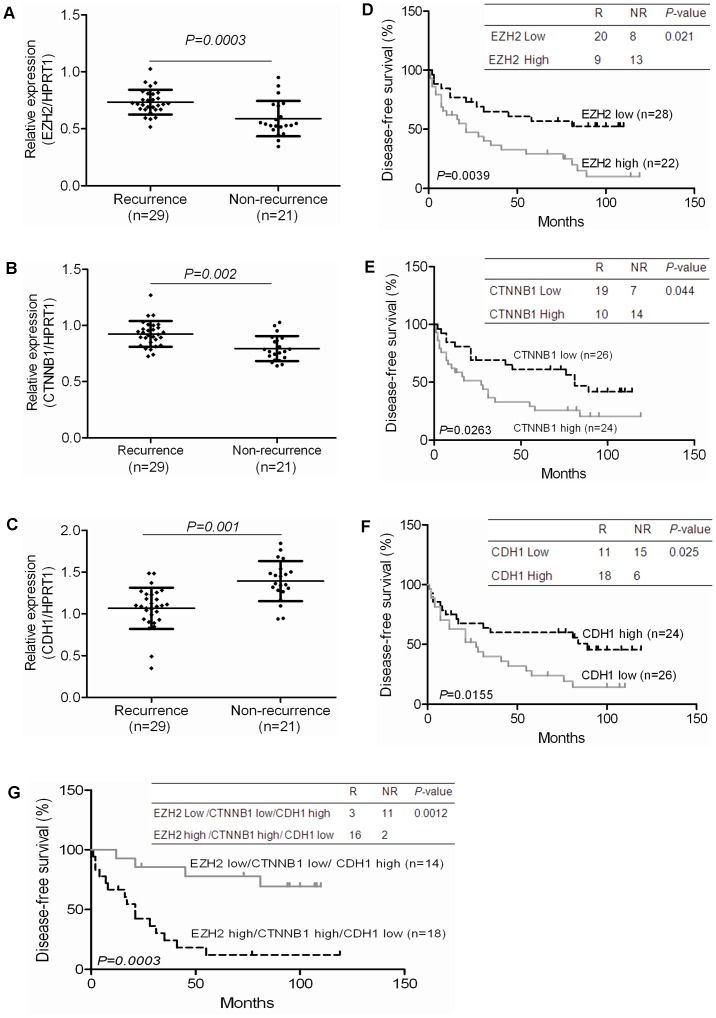
Expression of *EZH2, CTNNB1,* and *CDH1*, downstream targets of miR-214, is associated with early recurrent disease and survival of patients with HCC. The level of expression of *EZH2*, *CTNNB1,* and *CDH1* in the 50 HCC samples described in Figure 1 was analyzed by qRT-PCR and normalized by *HPRT1*. (A–C) The average expression level of *EZH2* and *CTNNB1* was significantly higher in samples from HCC patients with early recurrence (≤2 years) (n = 29) than patients with no recurrence over the same period (n = 21). In comparison, the average expression level of *CDH1* was significantly lower in in samples from HCC patients with early recurrence (C). (D–F) The median expression value obtained for *EZH2*, *CTNNB1*, and *CDH1* of the 50 samples studied was employed as the cut-off point and employed independently in Kaplan-Meier analysis to study disease-free survival rate. (G) Combining the expression of *EZH2*, *CTNNB1* and *CDH1* to predict tumor recurrence and disease-free survival rate using Kaplan-Meier analysis.

To validate *EZH2* and *CTNNB1* were indeed direct targets of miR-214, we tested the ability of miR-214 to recognize the 3′-UTR of *EZH2* and *CTNNB1* mRNA using a dual-luciferase reporter assay. The 3′-UTR sequence of *EZH2* and *CTNNB1* predicted to interact with miR-214 were synthesized and inserted into the XbaI and FseI sites of the pGL3 control vector (Promega, Madison, WI). Mutations were also been made into the predicted interacting sequences (Figure S3). These constructs were designated as pGL3-EZH2-3′UTR-wt, pGL3- EZH2-3′UTR-mut, pGL3-CTNNB1-3′UTR-wt and pGL3-CTNNB1-3′UTR-mut. Co-transfection of P-miR-214 with pGL3-EZH2-3′UTR-wt or pGL3-CTNNB1-3′UTR-wt in SK-HEP-1 cells significantly suppressed luciferase activity (Fig. 4A). The suppressive effect of P-miR-214 was abrogated with the pGL3-EZH2-3′UTR-mut and pGL3-CTNNB1-3′UTR-mut constructs which contained mutation introduced in the predicted miRNA-target interactions sequences, confirming that *EZH2* and *CTNNB1* were indeed direct downstream functional targets of miR-214 (Fig. 4A). Overexpression of miR-214 also inhibited the protein expression of EZH2 and CTNNB1 (Fig. 4B). Moreover, expression of E-cadherin, which often complexes with *CTNNB1*, was induced following the overexpression of miR-214 (Fig. 4B).

To corroborate the role of *EZH2* and *CTNNB1* in miR-214-mediated tumor cell invasion, we rescued the expression of *EZH2* and *CTNNB1* in miR-214 stable transfected HLE cells by transfecting a plasmid carrying wild-type *EZH2* (pLVTHM-EZH2) [16] or *CTNNB1* (pCI-CTNNB1, Addgene) [20]. The re-expression of *EZH2* and *CTNNB1* was validated by qRT-PCR (Figure S4). The cell invasion was partially rescued in miR-214 stable transfected HLE cells by reexpression of *EZH2* or *CTNNB1* (Figure S4). These data indicate that miR-214 inhibits cell invasion by inhibiting *EZH2* and *CTNNB1*. Corroborating with these results, expression of *EZH2*, *CTNNB1* and *CDH1* in a cohort of HCC patients studied in our laboratory previously by global gene expression profiling [4] also indicated that expression of both *EZH2* and *CTNNB1* are significantly up-regulated while *CDH1* is down-regulated in human HCC compared to adjacent histologically normal liver tissues (Figure S5). These results were further validated with the 20 paired human HCC tissue samples and the 10 samples of normal liver tissues by qRT-PCR described earlier (Fig. 4C–E).

### Silencing EZH2 or CTNNB1 or Functional Overexpressed CDH1 Suppressed HCC Cell Growth and Invasion

To investigate the roles of *EZH2*, *CTNNB1* and *CDH1* in HCC, we knocked-down the expression of *EZH2* and *CTNNB1* with shRNA and over-expressed *CDH1* with pcDNA3.1-CDH1 expression vector in SK-HEP-1 cells as described previously [16], [21], [22]. Silencing of *EZH2* and *CTNNB1* were confirmed by western blot (Fig. 5A (i) and 5B (i)). Silencing of *EZH2* significantly inhibited the growth and significant decreased the ability of SK-HEP-1 cells to invade (Fig. 5A (ii) and (iii)) while the knockdown of *CTNNB1* only significantly inhibited the growth of SK-HEP-1 cells (Fig. 5B (ii)). The over-expression of *CDH1* was confirmed by western blot (Fig. 5C (i)) and the over-expression of *CDH1* in SK-HEP-1 cells significantly inhibited their ability to invade (Fig. 5C (ii)). Furthermore, silencing of *EZH2* significantly decreased the expression of *CTNNB1* and induced *CDH1* expression (Fig. 5D). This observation is consistent with previous reports demonstrating that *EZH2* regulated the expression of *CTNNB1* and *CDH1* [23], [24].

### Silencing of miR-214 Increase EpCAM^+^ Stem-like Cell Population by Activating β-catenin Pathway

Previous studies have implicated that epithelial cell adhesion molecule (EpCAM) is an biomarker of HCC tumor-initiating cells with stem/progenitor cell features and EpCAM^+^ HCC cells were correlated with tumor progression and invasiveness [25]. Since EpCAM is a direct transcriptional target of the wnt-β-catenin canonical signaling pathway [26], [27], we have demonstrated that mR-214 can directly or indirectly modulate the expression of *CTNNB1* through *EZH2*, we decided to investigate the effect of silencing miR-214 on the EpCAM^+^ HCC tumor cell population. For this study, we firstly employed the construct miRZip-214 (System Biosciences) to knockdown miR-214 expression in miR-214-stable transfected HLE cells. The ability to knockdown of miR-214 expression by miRZip-214 construct was verified by qRT-PCR (Fig. 6A). Knocked-down of miR-214 by miRZip-214 specifically increased the expression of *EZH2* and β-catenin compared with miRZip-control transfection (Fig. 6B). Next, we overexpressed *EZH2* by transfecting the vector pLVTHM-EZH2 [16] to miR-214-stable transfected HLE cells. The overexpression of *EZH2* by pLVTHM-EZH2 also activated β-catenin while pLVTHM-control had no effect (Fig. 6C). Subsequent flow cytometric analysis showed that knocked-down of miR-214 or functional overexpressing *EZH2* induced an enrichment of EpCAM^+^ HLE cells (Fig. 6D (i) and (ii) respectively). These results suggest that miR-214 can modulate EpCAM^+^ stem-like cells by activating the β-catenin pathway in HCC cells.

### Expression of CTNNB1, EZH2 and CDH1 Associated with HCC Recurrence

We have previously established a gene expression profiling dataset on 50 HCC patients using Affymetrix Gene chips [4]. Of the 50 HCC patients studied, 29 gave early recurrent disease (<2 years) and 21 no recurrent disease developed till the last follow-up (non-recurrence). In the Fig. 1B, we demonstrated that miR-214 was significantly suppressed in samples of HCC patients with early recurrent disease, and high miR-214 expression was significantly associated with early recurrent disease and a relative poorer disease-free survival rate (Fig. 1C). To study the potential prognostic significance of *CTNNB1*, *EZH2*, the direct targets of miR-214 and *CDH1,* a downstream target in HCC recurrence, we further analyzed the expression level of *EZH2*, *CTNNB1*, and *CDH1* in this dataset. Besides miR-214 expression, it was observed that the upregulation of *EZH2* and *CTNNB1* and the down-regulation of *CDH1* significantly associated with early recurrent HCC disease and poor survival. Multivariate Cox regression analysis indicated that tumor recurrence, low-level of miR-214 and *CDH1*, and high-level of *EZH2* and *CTNNB1* were prognostic factors for early HCC recurrence and poor survival (Table S2). The expression of *CDH1* in HCC samples of patients with early recurrence was significantly lower while the expression of *EZH2* and *CTNNB1* was significantly higher in patients with early recurrence (Fig. 7A–7C). When the corresponding median value of the 50 samples studied was chosen as the cut-off point for high and low expression, Fisher’s exact test and Kaplan-Meier analysis showed that low-level *CDH1,* high *EZH2* and high *CTNNB1* expression was significantly associated with poor disease-free survival (Fig. 7D–7F). Combining *EZH2*, *CTNNB1* and *CDH1* improved synergistically the association with disease-free survival (Fig. 7G). These data suggest that the expression of miR-214 and its downstream targets are clinically useful indicators correlated with early recurrent HCC disease and survival.

## Discussion

The deregulation of miRNAs has been implicated in various human cancers including human hepatocellular carcinoma. Differential miRNA expression in tumor samples compared to normal samples or between groups of tumor samples with a favourable and poor clinical outcome have been used to generate miRNA signatures with potential prognostic and/or predictive value. Nevertheless, elucidating the molecular roles of aberrant miRNA expression in human HCC remains largely unexplored. In the current study, we have demonstrated the down-regulation of miR-214 is associated with the invasion and early recurrence of HCC. This finding extends earlier reports demonstrating the frequent down-regulation of miR-214 expression in human HCC tissues and cell lines [9], [10], [11], [12], [13]. More recently, ER stress has also been shown to negatively modulate the expression of the miR-199a/214 cluster to regulates tumor survival and progression in HCC [28]. Besides HCC, miR-214 has also been shown to be deregulated in human ovarian, cervical, and breast cancers [29], [30], [31], [32]. In ovarian cancer, miR-214 was shown to induce cell survival and cisplatin resistance by targeting the 3′-untranslated region (UTR) of *PTEN* to suppress its expression and resulting in the activation of the PI3K/Akt signaling pathway [29]. In cervical cancer, the ectopic expression of miR-214 could inhibit the proliferation, migration and invasive ability of HeLa cells by targeting *MEK3*, *JNK1* and Plexin-B1 [30], [31]. Studies have also reported that miR-214 contributes to the progression and metastasis of melanoma through the suppression of *TFAP2C* [33], [34]. These examples illustrate the importance of the proper execution of miR-214 for maintenance of cellular homeostasis and the optimal performance of cellular processes and miR-214 expression is often perturbed in human cancer.

The aberrant expression of miRNAs can be related to the metastatic capability of tumors, offer prognostic value as well as the identification of regulatory signaling pathways. Furthermore, miRNAs can coordinately modulate the expression of hundreds of target genes, mainly by negatively affecting mRNA stability and/or protein output. With this mode of gene expression control, a single miRNA can concomitantly influence multiple cellular programs under physiological and pathological conditions [35], [36], [37], [38]. Therefore, the prediction and identification of target genes is an important step toward deciphering the molecular roles of misregulated miRNAs. In this study, we have demonstrated that *CTNNB1* and *EZH2* are direct targets of miR-214. Since *CTNNB1* is also a downstream target of *EZH2*, hence *CTNNB1* can be directly or indirectly regulated though *EZH2* to modulate the β-catenin signalling pathway. We demonstrated that both *EZH2*, and *CTNNB1* were overexpressed in HCC patients with early recurrent disease. The regulation of the expression of the polycomb protein *EZH2* by miR-214 was firstly observed in skeletal muscle and embryonic stem cells (29). Subsequently, the overexpression of *EZH2* has been reported in several types of cancer including prostate, breast, bladder, gastric, lung, and HCC [39]. Most recently and in agreement with our present results, it has been reported that the reduction of miR-214 expression in breast cancer cells associated with increase in cell proliferation, invasion, and accumulation of the polycomb EZH2 methyltransferase [32] and that EZH2 protein expression can be a sensitivity diagnostic biomarker for HCC [40]. In the present study, we have validated these observations and further demonstrated that suppression of miR-214 expression in HCC can modulate the β-catenin signalling pathway by activating *CTNNB1* and *EZH2* and down-regulating *CDH1* expression. Additionally, our clinical data showed that low-level of miR-214 and *CDH1* and high-level of *EZH2* and *CTNNB1* were significantly associated with early recurrent HCC disease and can be predictors of comparatively reduction in disease-free survival (Fig. 7).

Despite several reports have implicated the aberrant activation and mutation of *CTNNB1*, to the best of our knowledge, there has been no report to suggest that *CTNNB1* to be a direct target of miR-214 in HCC [41], [42]. In this study, we demonstrated the expression of *CTNNB1* was significantly up-regulated in HCC tumors compared to adjacent histologically normal liver tissues. The silencing of *CTNNB1* expression significantly inhibits HCC cell growth and induced the expression of E-cadherin. Previous studies have established that cadherin adhesion is necessary for cell-cell junctional complex assembly [43], [44]. Cadherins associate with a growing number of membrane cytoskeletal proteins termed the cadherin/catenin complex [45]. Catenins are the best-characterized cadherin binding proteins, and their binding is required for cadherin function. Loss of E-cadherin-beta-catenin adhesion has been shown to be associated with the progression of many epithelial malignancies and it is linked to tumor metastasis and poor clinical prognosis [23]. Herein, we also showed that the expression of E-cadherin (*CDH1*) is significantly down-regulated in human HCC tissues and the functional overexpression of E-cadherin in HCC cells significantly inhibited cell invasion. Moreover, the expression of *CTNNB1* and *CDH1* were significantly associated with early tumor recurrence.

Cancer stem cells (CSCs) are cells within a tumor that possess the capacity to self-renew and maintain tumor-initiating capacity through differentiation into the heterogeneous lineages of cancer cells that comprise the whole tumor [46], [47]. Recent evidence suggests that epithelial cancers, including HCC are driven by a small sub-population of CSCs. For HCC, it has been reported that EpCAM can be a marker for the putative hepatic CSCs [25]. One of the characteristics of CSCs is their ability to form floating spheroids under anchorage-independent conditions in a serum-free defined media. Colonospheres formed in vitro exhibited higher expression of colon CSCs markers including EpCAM, and also exhibited the ability to form spheroids under extreme limiting dilution, indicating the predominance of CSCs in colonospheres [48]. Additionally, colonospheres showed reduced membrane bound β-catenin and the down-regulation of phosphorylated β-catenin. Since miR-214 can regulate the β-catenin pathway directly or indirectly via *EZH2* to target β-catenin [48], we have investigated the potential role of miR-214 in regulating hepatic EpCAM^+^ CSCs. It can be demonstrated by flow cytometric analysis that silencing of miR-214 or functional overexpressing *EZH2* induced an enrichment of EpCAM^+^ HCC cells and suggesting that miR-214 can modulate EpCAM^+^ stem-like cells by activating the β-catenin pathway.

In summary, our data showed that miR-214 can provide tumor suppression function in hepatocarcinogenesis through modulating the expression of *EZH2*, *CTNNB1* and *CDH1*. The reduction in the expression level of miR-214 during hepatocarcinogenesis resulted in the up-regulation of EZH2 and β-catenin and the down-regulation of E-cadherin. The ectopic expression of miR-214 in HCC cell lines suppressed cell growth, invasion and stem-like traits *in vitro* and tumorigenicity *in vivo* through the inhibition of β-catenin signaling. Hence, restoration the expression of miR-214 in HCC can be explored as an alternative therapeutic strategy against HCC.

## Supporting Information

Figure S1 **Expression of miR-214 in 20 paired of HCC tumor tissues was significantly lower compared to 20 matched histologically normal tissues (** ***P*** **<0.01).** The 2^–ΔΔCt^ of the values was calculated by normalization to the values obtained with the “matched normal” tissues as the “reference”.
(TIF)

Figure S2 **Expression of miR-214 in transfected or stable cells.** (A, C) Images obtained after P-miR-control and P-miR-214 transfection in HLE and SK-HEP-1 cells. (B, D) Relative level of expression of miR-214 after transfection with P-miR-214 in HLE and SK-HEP-1 cells 48 h after transfection. (E) Images of SK-HEP-1-miR-control and SK-HEP-1-miR-214 stable cells. (F) Relative level of expression of miR-214 in SK-HEP-1-miR-control and SK-HEP-1-miR-214 stable cells.
(TIF)

Figure S3 **(A) The modified pLL3.7 plasmid structure and the insert sequence of hsa-miR-214.** (B) The map of pGL3 control vector. The 3′-UTR sequence or a mutated sequence were synthesized and inserted into the XbaI and FseI sites of the pGL3 control vector. (C,D) Rnahybrid analysis of miR-214 and EZH2-3′UTR or CTNNB1–3′UTR by RNAhybrid 2.2 predicted target sequences of miR-214 within the 3′-UTR of EZH2 (C) or CTNNB1 mRNA (D).
(TIF)

Figure S4 **Re-expression of EZH2 and CTNNB1 rescues miR-214-related functions.** (A,B) The re-expression of EZH2 or CTNNB1 in miR-214 stable transfected HLE cells was validated by qRT-PCR. (C,D) The cell invasion was partially rescued in miR-214 stable transfected HLE cells by re-expression of EZH2 or CTNNB1.
(TIF)

Figure S5 **Expression of EZH2, CTNNB1 and CDH1 in human HCC samples.** (A–C) The expression of EZH2, CTNNB1 and CDH1 was from a previously published HCC microarray database and presented by dot plot analysis. The microarray data for the HCC tumors, matched normal and histologically normal liver tissues have been previously deposited in the European Bioinformatics Institutes of the European Molecular Biology Laboratory database (http://www.ebi.ac.uk/arrayexpress/) and are accessible through ArrayExpress public database with accession numbers E-MEXP-84. EZH2 (A) and CTNNB1 (B) were significantly up-regulated while CDH1 (C) was down-regulated in human HCC tissue samples.
(TIF)

Table S1 **Primers for qRT-PCR analysis.**
(DOCX)

Table S2 **Univariate and multivariate disease-free survival analysis for the 50-samples real-time PCR dataset, based on known clinical parameters.**
(DOCX)
