# Correction: MiR-214 Targets β-Catenin Pathway to Suppress Invasion, Stem-Like Traits and Recurrence of Human Hepatocellular Carcinoma

**DOI:** 10.1371/annotation/1be2a62e-45a1-4c13-9a8d-f265005a21e0

**Published:** 2012-09-28

**Authors:** Hongping Xia, London Lucien P. J. Ooi, Kam M. Hui

There were errors in the Funding statement.

The correct statement is "This work was supported by grants from the SingHealth Foundation (http://www.singhealth.com.sg), National Medical Research Council (http://www.nmrc.gov.sg), Biomedical Research Council of Singapore and The Singapore Millennium Foundation. The funders had no role in study design, data collection and analysis, decision to publish, or preparation of the manuscript. 

