# Supplementary figures and images for: MiR-214 Targets β-Catenin Pathway to Suppress Invasion, Stem-Like Traits and Recurrence of Human Hepatocellular Carcinoma
Source: PLoS One. 2012 Sep 4;7(9):e44206. doi: 10.1371/journal.pone.0044206 (PMC3433464; doi:10.1371/journal.pone.0044206)

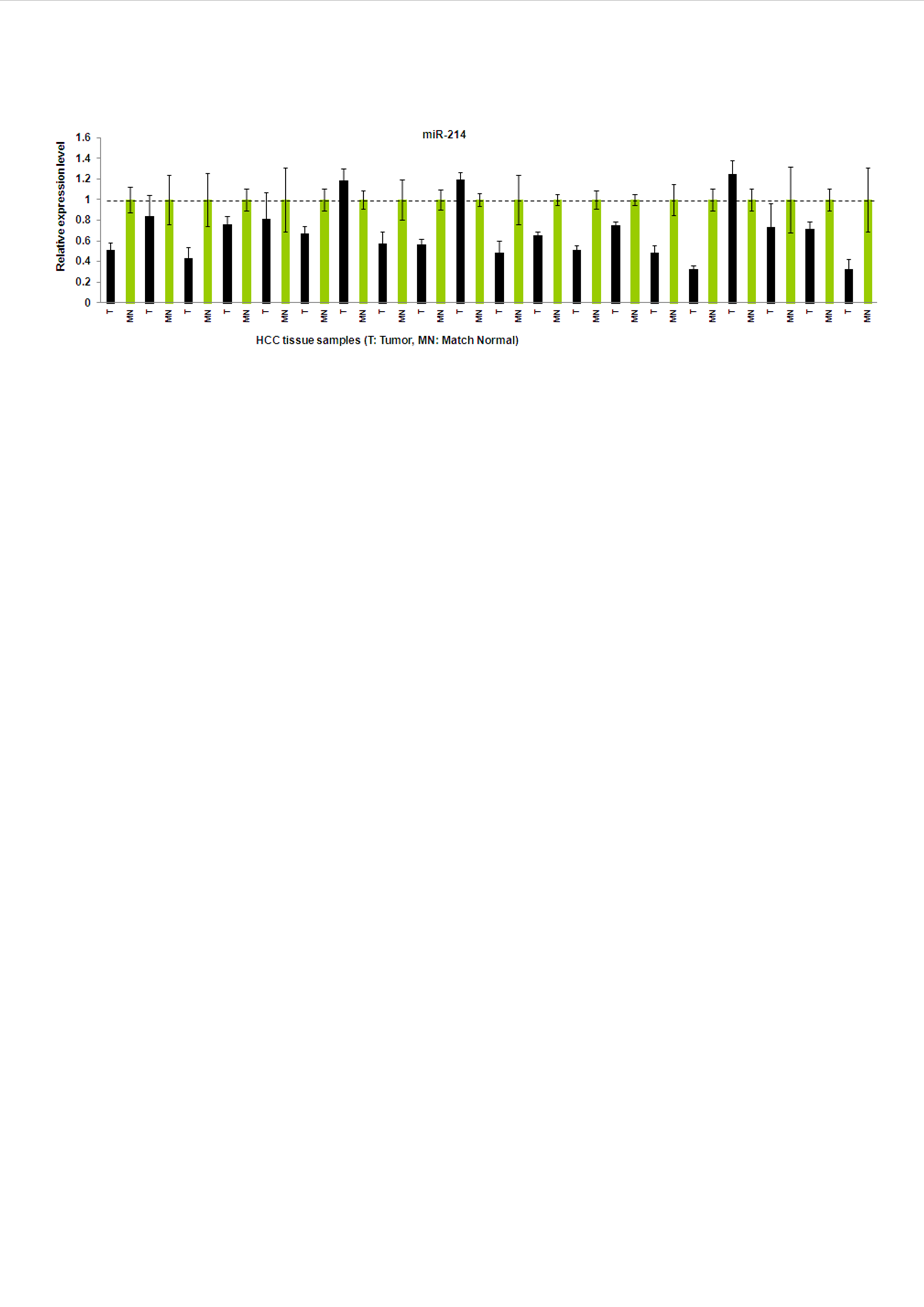

Supplement: Figure S1 — Expression of miR-214 in 20 paired of HCC tumor tissues was significantly lower compared to 20 matched histologically normal tissues ( P <0.01). The 2–ΔΔCt of the values was calculated by normalization to the values obtained with the “matched normal” tissues as the “reference”. (TIF) [file pone.0044206.s001.tif]

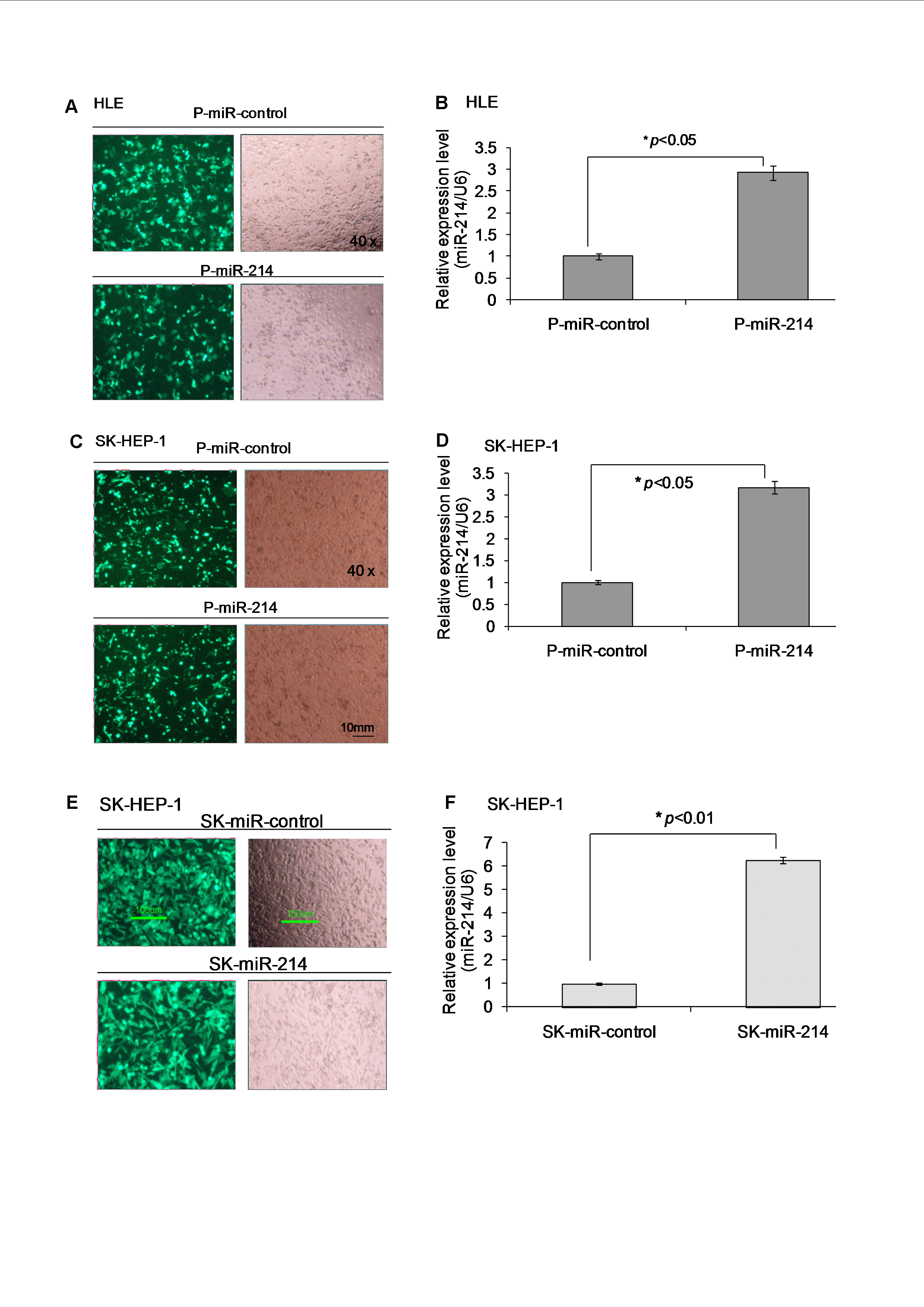

Supplement: Figure S2 — Expression of miR-214 in transfected or stable cells. (A, C) Images obtained after P-miR-control and P-miR-214 transfection in HLE and SK-HEP-1 cells. (B, D) Relative level of expression of miR-214 after transfection with P-miR-214 in HLE and SK-HEP-1 cells 48 h after transfection. (E) Images of SK-HEP-1-miR-control and SK-HEP-1-miR-214 stable cells. (F) Relative level of expression of miR-214 in SK-HEP-1-miR-control and SK-HEP-1-miR-214 stable cells. (TIF) [file pone.0044206.s002.tif]

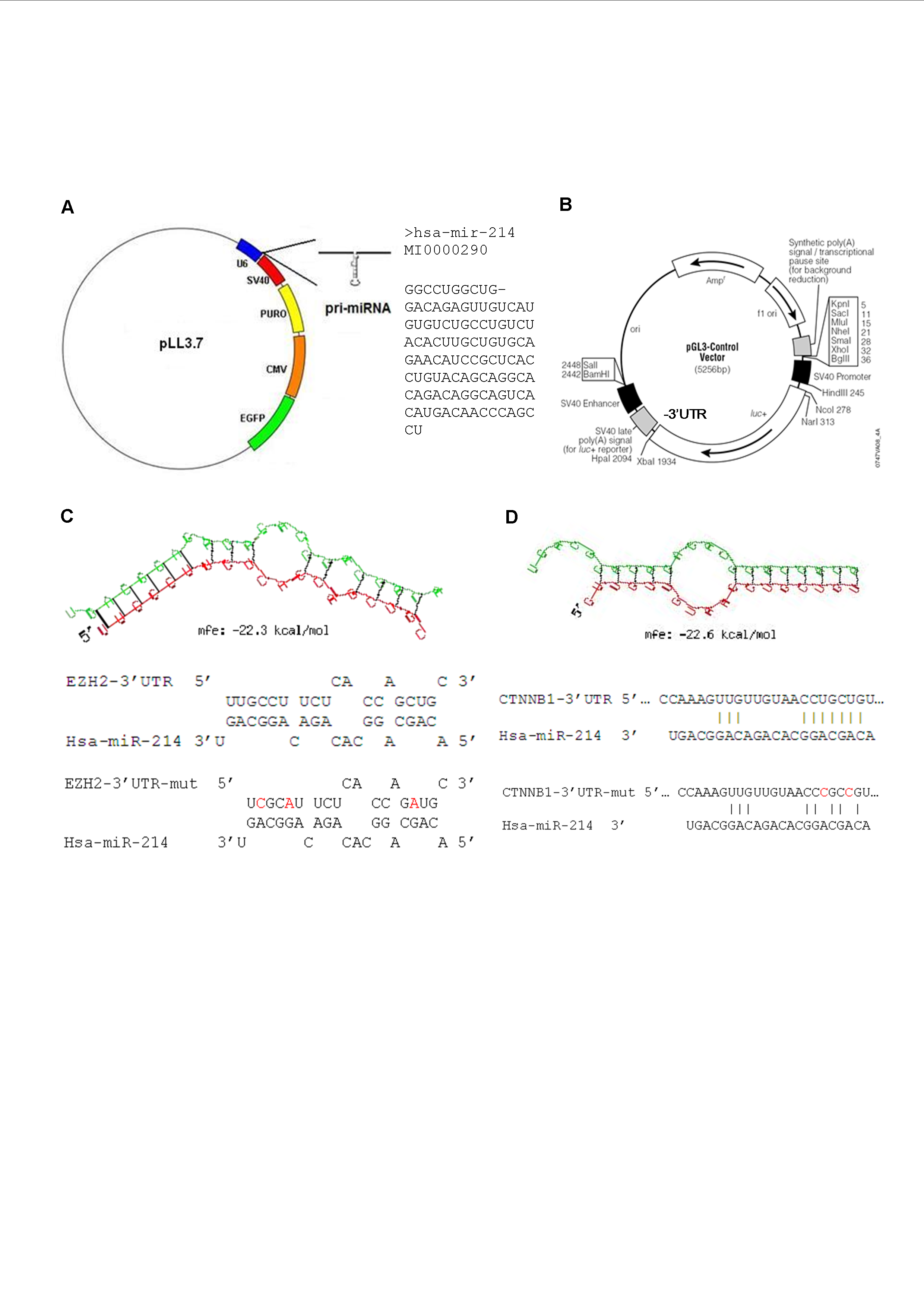

Supplement: Figure S3 — (A) The modified pLL3.7 plasmid structure and the insert sequence of hsa-miR-214. (B) The map of pGL3 control vector. The 3′-UTR sequence or a mutated sequence were synthesized and inserted into the XbaI and FseI sites of the pGL3 control vector. (C,D) Rnahybrid analysis of miR-214 and EZH2-3′UTR or CTNNB1–3′UTR by RNAhybrid 2.2 predicted target sequences of miR-214 within the 3′-UTR of EZH2 (C) or CTNNB1 mRNA (D). (TIF) [file pone.0044206.s003.tif]

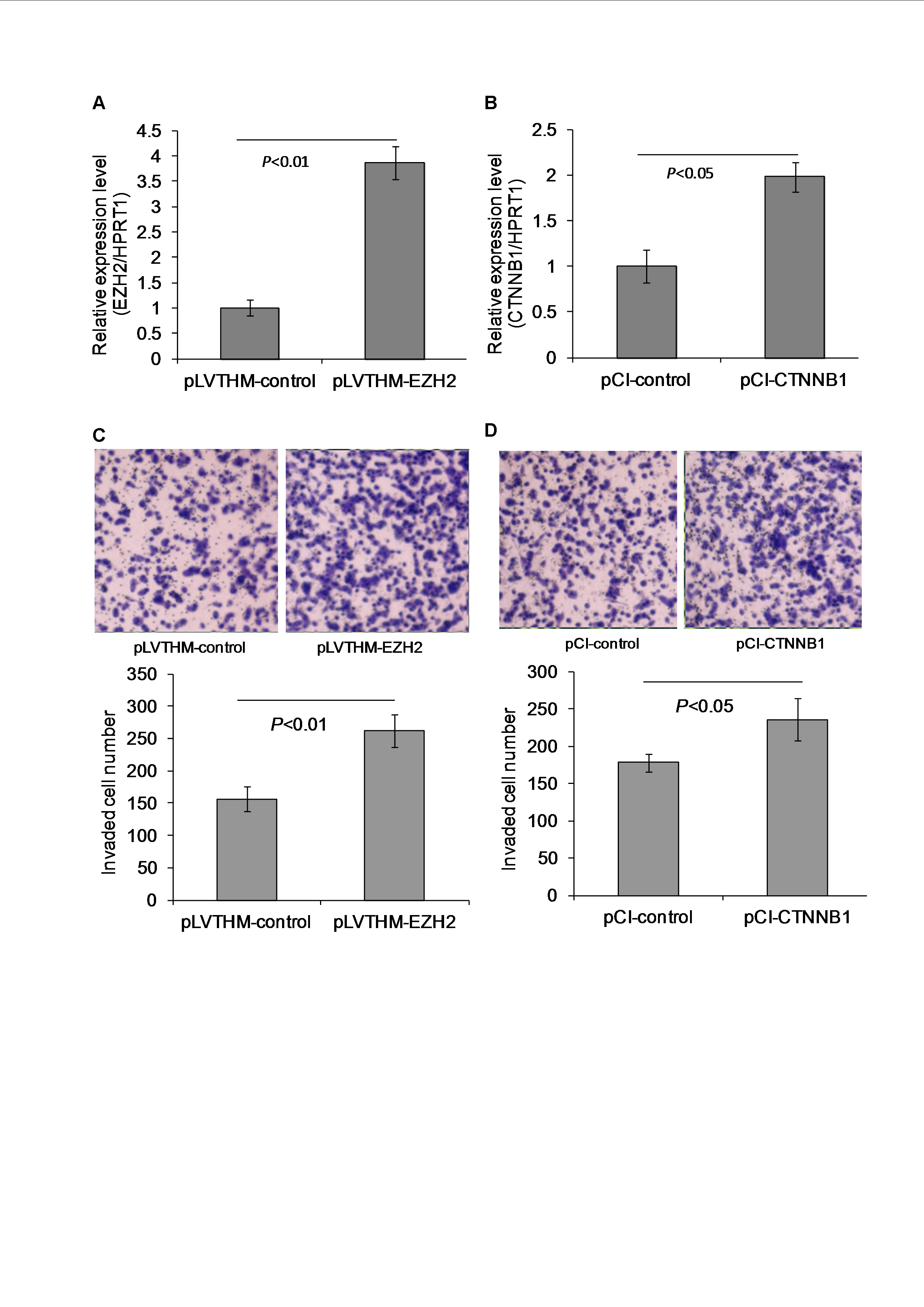

Supplement: Figure S4 — Re-expression of EZH2 and CTNNB1 rescues miR-214-related functions. (A,B) The re-expression of EZH2 or CTNNB1 in miR-214 stable transfected HLE cells was validated by qRT-PCR. (C,D) The cell invasion was partially rescued in miR-214 stable transfected HLE cells by re-expression of EZH2 or CTNNB1. (TIF) [file pone.0044206.s004.tif]

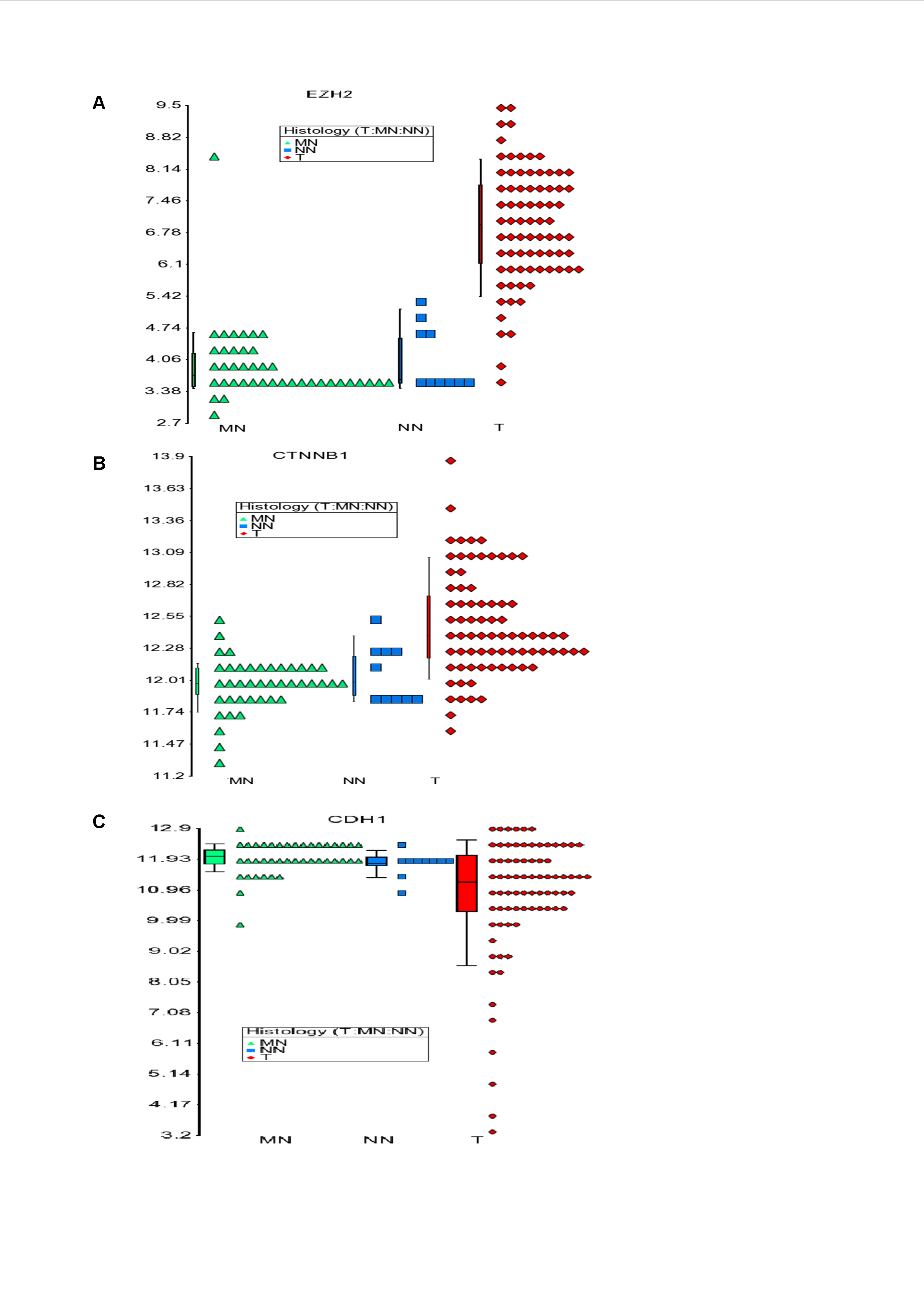

Supplement: Figure S5 — Expression of EZH2, CTNNB1 and CDH1 in human HCC samples. (A–C) The expression of EZH2, CTNNB1 and CDH1 was from a previously published HCC microarray database and presented by dot plot analysis. The microarray data for the HCC tumors, matched normal and histologically normal liver tissues have been previously deposited in the European Bioinformatics Institutes of the European Molecular Biology Laboratory database (http://www.ebi.ac.uk/arrayexpress/) and are accessible through ArrayExpress public database with accession numbers E-MEXP-84. EZH2 (A) and CTNNB1 (B) were significantly up-regulated while CDH1 (C) was down-regulated in human HCC tissue samples. (TIF) [file pone.0044206.s005.tif]
