# Supplementary material for: MiR-214 Targets β-Catenin Pathway to Suppress Invasion, Stem-Like Traits and Recurrence of Human Hepatocellular Carcinoma
Source: PLoS One. 2012 Sep 4;7(9):e44206. doi: 10.1371/journal.pone.0044206 (PMC3433464; doi:10.1371/journal.pone.0044206)
Supplement: Table S1 — Primers for qRT-PCR analysis. (DOCX) [file pone.0044206.s006.docx]

**Table S1.** Primers for qRT-PCR analysis

| **Gene** | **Forward primer (5’-3’)** | **Reverse primer (5’-3’)** |
| --- | --- | --- |
| EZH2 | GGTTCAGACGAGCTGATGAAG | CGCTGTTTCCATTCTTGGTT |
| CTNNB1 | GAAACGGCTTTCAGTTGAGC | CTGGCCATATCCACCAGAGT |
| CDH1 | AGTGGGCACAGATGGTGTGA | TAGGTGGAGTCCCAGGCGTA |
| HPRT1 | TGACACTGGCAAAACAATGCA | GGTCCTTTTCACCAGCAAGCT |
| Hsa-miR-214 | CAGGCACAGACAGGCAGT | Universal qPCR Primer (Invitrogen) |
| U6 | CTCGCTTCGGCAGCACA | Universal qPCR Primer (Invitrogen) |
