# Supplementary material for: MiR-214 Targets β-Catenin Pathway to Suppress Invasion, Stem-Like Traits and Recurrence of Human Hepatocellular Carcinoma
Source: PLoS One. 2012 Sep 4;7(9):e44206. doi: 10.1371/journal.pone.0044206 (PMC3433464; doi:10.1371/journal.pone.0044206)
Supplement: Table S2 — Univariate and multivariate disease-free survival analysis for the 50-samples real-time PCR dataset, based on known clinical parameters. (DOCX) [file pone.0044206.s007.docx]

**Table S2.** Univariate and multivariate disease-free survival analysis for the 50-samples real-time PCR dataset, based on known clinical parameters

| Clinical parameters | Case number | Univariate *P*-value | Multivariate *P*-value |
| --- | --- | --- | --- |
| Gender (M vs. F) | 43/7 | 0.117 |  |
| Age (>50 vs. ≤50 years) | 35/15 | 0.245 |  |
| AFP (>400 vs. ≤400 ng/mL) | 15/35 | 0.113 |  |
| Total bilirubin (>25 vs. ≤ 25 U/L) | 5/45 | 0.151 | 0.584 |
| Child-Pugh score (A vs. B) | 39/11 | 0.047 |  |
| Tumor size (> 5 vs. ≤ 5 cm) | 25/25 | 0.098 |  |
| Satellite nodule (>1 vs. 1) | 8/42 | 0.468 |  |
| Cirrhosis (Yes vs. No) | 29/21 | 0.311 |  |
| AJCC tumor staging (I&II vs.) | 42/8 | 0.164 |  |
| Venous infiltration (VI vs. NI) | 20/30 | 0.028 | 0.649 |
| Recurrence (R vs. NR) | 29/21 | 0.000 | 0.017 |
| miR-214 (low vs. high) | 22/28 | 0.000 | 0.004 |
| EZH2 (low vs. high) | 28/22 | 0.0039 | 0.031 |
| CTNNB1 (low vs. high) | 26/24 | 0.0263 | 0.037 |
| CDH1 (low vs. high) | 26/24 | 0.0155 | 0.019 |

Univariate analysis performed using the log-rank test. Multivariate analysis performed using the Cox regression model and Wald’s test. The expression levels of miR-214, EZH2, CTNNB1 and CDH1 were examined by real-time quantitative PCR and normalized by U6 or HPRT1, respectively. The median value of all 50 samples was chosen as the cut-off point.
